# Immunostimulatory activity of water-extractable polysaccharides from *Cistanche deserticola* as a plant adjuvant *in vitro* and *in vivo*

**DOI:** 10.1371/journal.pone.0191356

**Published:** 2018-01-23

**Authors:** Ailian Zhang, Xiumei Yang, Quanxiao Li, Yu Yang, Gan Zhao, Bin Wang, Daocheng Wu

**Affiliations:** 1 Xinjiang Key Lab of Biological Resources and Genetic Engineering, College of Life Science and Technology, Xinjiang University, Urumqi, Xinjiang, China; 2 Key Lab of Medical Molecular Virology, School of Basic Medical Science, Shanghai Medical College, Fudan University, Shanghai, China; 3 College of Life Science and Technology, Xi’an Jiaotong University, Xian, Shanxi, China; National Institute of Animal Biotechnology, INDIA

## Abstract

A safe and effective vaccine adjuvant is important in modern vaccines. Various Chinese herbal polysaccharides can activate the immune system. *Cistanche deserticola* (CD) is a traditional Chinese herb and an adjuvant candidate. Here, we confirmed that water-extractable polysaccharides of CD (WPCD) could modulate immune responses *in vitro* and *in vivo*. In a dose-dependent manner, WPCD significantly promoted the maturation and function of murine marrow-derived dendritic cells (BM-DCs) through up-regulating the expression levels of MHC-II, CD86, CD80, and CD40, allogenic T cell proliferation, and the yields of IL-12 and TNF-α via toll-like receptor4 (TLR4), as indicated by *in vitro* experiments. In addition, its immunomodulatory activity was also observed in mice. WPCD effectively improved the titers of IgG, IgG_1_ and IgG_2a_ and markedly enhanced the proliferation of T and B cells, the production of IFN-γ and IL-4 in CD4^+^ T cells and the expression level of IFN-γ in CD8^+^ T cells better than Alum. Furthermore, WPCD could markedly up-regulate the expression levels of CD40 and CD80 on DCs in spleen and down-regulate the Treg frequency. The study suggests that polysaccharides of *Cistanche deserticola* are a safe and effective vaccine adjuvant for eliciting both humoral immunity and cellular immunity by activating DCs via TLR4 signaling pathway.

## Introduction

Vaccines are important for controlling or preventing diseases. Newly generated and current developing vaccines are highly purified recombinant antigens with a higher safety, but purified antigens cannot stimulate a satisfactory immune response compared with attenuated or inactivated pathogen preparations. With the aid of adjuvants, vaccines can induce persistent immune responses [1,2]. New powerful adjuvants are widely concerned in both vaccine development and human health. To date, various adjuvants have been identified and extensively studied. These adjuvants endow vaccines with several advantages, including decreasing the required amount of antigens, minimizing the number of immunizations required for normal immune responses, and inducing more rapid, broader, and stronger immune responses [3–5]. Although many potent adjuvants have been developed, such as lipolysaccharide (LPS) and Freund’s complete adjuvant (FCA), they are not widely applied due to their toxicity. Hence, only a few adjuvants, such as Alum, are licensed for clinical use [6]. Overall, more effective and safer adjuvants should be developed to promote the better prophylactic and therapeutic vaccines against infectious and noninfectious diseases.

Safety is an important consideration factor in the development of adjuvants. Chinese herbaceous constituents include nutrients and important active composites, such as phenolic compounds and polysaccharides, which can act as powerful immunostimulants [7–9]. Among them, polysaccharides from traditional Chinese herbs have been widely explored due to their immunostimulatory activities and low toxicity. For example, inulin is an immune adjuvant without the toxicity of other adjuvants such as FCA. Advax^TM^ delta inulin adjuvant also has successfully enhanced the immunogenicity of vaccine [10,11]. Astragalus, also known as Huangqi in Chinese and Radix Astragali in Latin, is widely distributed throughout the world. Polysaccharide is one of its major active ingredients responsible for the immunomodulatory activity. Experimental studies have demonstrated that Astragalus polysaccharide possessed strong immunomodulatory effects both *in vitro* and *in vivo* [12, 13]. Lycium barbarum polysaccharides as vaccine adjuvant also showed good improvements and stimulating effects [14, 15]. These studies suggested that Chinese herbral polysaccharides were ideal candidates for adjuvant development.

*Cistanche deserticola Y*. *C*. *Ma* (CD, "Rou Cong Rong" in Chinese) is a valuable traditional Chinese herb and commonly considered as “Ginseng of the deserts” because of its superior tonic effects. It is distributed in arid or semi-arid regions in Xinjiang. In China, its dried fleshy stem has been used as a tonic food for hundreds of years [16, 17]. For many years, boiled CD has been used as a tonic food to treat overstrain-induced impairment, suggesting that it is safe for oral administration. This plant is widely concerned due to its broad medicinal functions. Recent phytochemical and pharmacological studies demonstrated that polysaccharides were the main biologically active components and had various biological effects, including the immunomodulatory activity, antioxidant effect, and anti-inflammatory effects [18–21]. However, the immune enhancement activity of water-extractable polysaccharides from *C*. *deserticola* in Xinjiang was seldom reported.

It is well known that DCs are important antigen presenting cells (APCs) providing signals required for initiating immune responses and modulating innate and adaptive cells. Some adjuvants improving antigen uptake by DCs can increase co-stimulatory or MHC molecules and enhance the immunity. When DCs maturation starts, more co-stimulatory molecules and cytokines are produced and DCs show distinct phenotypes [22, 23].

In this study, we firstly exploited whether WPCD could promote activation of DCs via TLR4 signaling pathway *in vivo*. Since DCs were the link between innate and adaptive immune responses, we hypothesized that DCs activation stimulated by WPCD would influence the outcome of adaptive immunity. We examined the specific immune response to ovalbumin (OVA) in mice treated with WPCD, including titers of IgG, IgG_1_ and IgG_2a_ subclass, T- and B- proliferation, and the production of cytokine. We investigated whether WPCD treatment would increase the activity of DCs and Treg cells in the spleen of these mice. Our findings proved the potential immunomodulatory activity of natural polysaccharides from *C*. *deserticola* and expanded its application scope.

## Materials and methods

### Animals

Eight- to ten-week old C57BL/6, BALB/c or ICR female mice were purchased from the First Hospital of Xinjiang Medical University (Urimuqi, Xinjiang, China). The mice were housed under pathogen-free conditions according to the guidelines of the Animal Care and Use Committee (ACUC) of Xinjiang University for Animal Health and Wellbeing. The animal experiment procedures had been approved by the AUCC of Xinjiang University.

### Extraction of aqueous extracts

*C*. *deserticola* is a plant in Xinjiang Province in China. Crude polysaccharide was obtained through water extraction and ethanol precipitation. Briefly, 100 g of dried *C*. *deserticola* was ground into powder and then filtered. Powder was refluxed with petroleum ether at room temperature repeatedly and then refluxed with anhydrous ethanol for 1 h to remove colored ingredients and lipids. Residues were extracted with boiling water for three times and the extracts were pooled together, centrifuged at 4000 rpm for 10 min, and refluxed at 60°C for 4 h. Four times of volumes of 95% ethanol was added into the solution in order to precipitate crude polysaccharides. The crude polysaccharides were re-dissolved in distilled water and treated with Sevage reagent to remove proteins. Extracts were dissolved in PBS and sterilized through 0.22-μm filter. Finally, the total sugar content of WPCD was 59.58%, as indicated by the phenol–sulfuric acid analysis [24].

### Generation of DCs

BM-DCs were generated according to the previously described method with slightly modifications [25]. Bone marrow DCs from C57BL/6 mice were flushed out with RPMI-1640 complete medium (Gibco) supplemented with 10% fetal calf serum (Hyclone). The collected cells were re-suspended in RPMI-1640 medium with 50 μM β-mercaptoethanol (Sigma, St Louis, MO) and 20 ng/mL murine GM-CSF (Peprotech, Rocky Hill, NY) and incubated at 37°C in 5% CO_2_ atmosphere. Half of the culture medium was replaced by fresh medium containing GM-CSF every 1–2 days. Cells were collected on Day 6, treated with different doses of WPCD, LPS (100 ng/mL) (Sigma, St Louis, MO) for 24 h and then assessed by flow cytometry.

### Detection of maturation of DCs and T cell activation *in vitro*

*In vitro* maturation effect of WPCD on BM-DCs was evaluated based on phenotypic analysis results by FACs. On Day 7, BM–DCs from C57BL/6 were induced in the presence of GM-CSF and then treated with different concentrations of WPCD (0.01, 0.02, 0.05, 0.1 and 0.2 mg/mL) for 12 h in triplicate. LPS (100 ng/mL) was used as a positive control. After BM-DCs were washed in PBS and Fc Block (BD Biosciences, CA) was used to prevent nonspecific antibody binding, cells were stained in PBS by using the appropriate FITC-, PE-, or APC-conjugated CD40, CD11c, CD86, CD80, and MHC-II antibodies (BD) for 20 min at 37°C. The stained cells were detected by FACs Calibur (BD). The data analysis was carried out with FlowJo software (Tree Star).

In DCs maturation experiment *in vitro*, cell culture supernatants were also collected to analyze IL-12 and TNF-α by using cytokine assay kits (Boster, Wuhan, China) according to the manufacturer’s instructions. The absorbance at 450 nm was measured with an ELISA plate reader (Bio-Rad, USA). Cytokine quantities in the samples were calculated with standard curves of recombinant cytokines according to the regression linear method.

To test their allogenic stimulatory activity, the experiment of mixed lymphocyte reaction (MLR) was performed according to the previous method [26] by MTT (Sigma, St Louis, MO) assay. Briefly, BM-DCs from C57BL/6 mice used as stimulator cells were recovered on Day 7 in RPMI-1640 medium plus GM-CSF and treated with various concentrations of WPCD for 12 h at 37°C. Cells were washed and re-suspended in RPMI-1640 medium before plating into the 24-well plate. Singleplenocyte suspensionas responder cells were isolated from BALB/c mice. BM-DCs were mixed with splenocytes according to the ratios of 1:5 and 1:10. Cells were cultured at 37°C in 5% CO_2_ atmosphere for three days in triplicate. LPS treatment was used as a positive control. Cultures were subsequently added with 20 μL of MTT for the final 4-h cultivation. The absorbances at the measuring wavelength (490 nm) and the reference wavelength (650 nm) were measured for T cell proliferation analysis. All samples were measured against a background control.

### Treatment by TLR4 inhibitor *in vitro*

BM-DCs were pre-treated with 5 μM TAK-242 (inhibitor of TLR4, Medchemexpress Inc. USA) for 1 h and then co-cultured with various concentrations of WPCD (100 and 200 μg/mL) for 12 h in the presence or absence of Golgi Stop in triplicate. In the positive control, LPS was added. Cells and supernatants were detected by FACs for analyzing surface markers CD86 and CD40 and by ELISA for analyzing cytokines TNF-a and IL-12, respectively.

### Immunization protocol

#### Acute toxicity test

In oral acute toxicity test in this study, 40 healthy adult ICR mice were used. ICR mice were fasted (food but not water overnight) before dosing. WPCD was orally administered respectively in the doses of 0, 50, 500 or 5000 mg/kg body weight to each animal for 7 days. Saline-treated and alum-treated mice were included in the control group. The animals were observed daily within 14 consecutive days. The mice were individually observed to record mortality and clinical signs. In addition, body weights and their food and water consumption were measured throughout the experiment. On Day 14, the spleen or thymus index was calculated as: (spleen or thymus weight/bodyweight) × 10.

#### Detection of WPCD immunomodulatory activity *in vivo*

To investigate whether WPCD had the immunomodulatory activity, ovalbumin (OVA) (Sigma) was used as the model antigen and female ICR mice were randomly separated into 7 groups (negative control group (0.9% NaCl and WPCD 400 μg) and the positive control (Alum 200 μg with OVA)). The mice were administered subcutaneously twice with 10 μg OVA alone or WPCD with OVA according to the dose of 20, 100 or 400 μg with a 2-week interval. Blood samples and spleen were obtained after the booster vaccination in order to detect IgG titers, the IgG subclasses, splenocyte proliferation and cytokine, DCs surface markers, and Treg frequency.

### Detection of OVA-specific antibodies

OVA-specific IgG titers and subclasses were examined by ELISA according to the previous method [27]. ELISA plates (Nunc, Thermo Fisher Scientific) were coated overnight and then blocked. Serum samples were diluted serially for IgG titer analysis or the detection of IgG_1_ and IgG_2a_ (Southern Biotech, Inc.). The plates were then incubated with PBST containing HRP-conjugated anti-mouse IgG, IgG_1_, and IgG_2a_ for 1 h at 37°C. The colorimetric reaction was developed with tetramethylbenzidine (TMB) and then the absorbances at 450 nm/655 nm was measured and expressed as optical density (OD) units.

### Detection of splenocyte proliferation and cytokine

Splenocyte proliferation was determined by the MTT assay. On Day 21 after the first vaccination, single splenocyte suspension was obtained. Cells were cultured in RPMI-1640 medium in 96-well plates according to the concentration of 1×10^6^ cells/well in triplicate. The cultures were stimulated with OVA (final concentration 10 μg/mL), ConA (final concentration 5 μg/mL) (Sigma, St Louis, MO), and LPS (final concentration 100 ng/mL) for 48 h at 37°C. Cells were cultured for 48 h and 20 μL (5 mg/mL) of MTT (Sigma, St Louis, MO) was added into each well and incubated for another 4 h. After adding 50 μL of DMSO into each well to stop the color development (Sigma), plates were read at 570 nm by a microtiter plate reader (Bio-Rad, CA, USA). Splenocyte proliferation was expressed as stimulation index (SI), which was the OD_570 nm_ ratio of a stimulated well to an unstimulated well.

The yields of IL-4 and IFN-γ in T cells were measured by intracellular cytokine staining according to a published protocol with minor modifications [27, 28]. Single splenocyte suspension was prepared on Day 21 after the first vaccination. After red blood cell lysis, the splenocyte (2×10^6^ cells/mL) were incubated with OVA (10 μg/mL) for 4-h stimulation and Golgi stop (BD) was added for 12 h incubation, PMA as positive control. Cells were collected, washed with PBS, stained with CD4-FITC/CD8-FITC, and fixed and permeabilized with Cytofix/Cytoperm kit (BD) according to the manufacturer instructions. Intracellular cytokine staining was performed with the appropriate concentration of IL-4-PE or IFN-γ-APC antibodies at 4°C for 20 min. Stained cells were detected by FACs. Data analysis was carried out in the FlowJo.

### Assessment of DCs maturation and Treg cells in the spleen

For the analysis of DCs maturation from splenocyte in mice, cell surface staining was performed with CD11c-PE, CD40-FITC, and CD80-APC antibodies. On Day 3 after the first vaccination, single splenocyte suspension (1×10^6^ cells/mL) was obtained and cells were subjected to double staining. The fluorescent intensities were measured by the FACs Calibur and the measured data were analyzed by FlowJo.

In order to observe whether WPCD could down-regulate the frequency of CD4^+^CD25^+^Foxp3^+^ Treg cells. Single splenocyte suspension was prepared on Day 7 after the second vaccination. The splenocyte (2×10^6^ cells/mL) was subjected to cell surface staining with CD4-APC antibody, followed by nuclear cytokine staining with the appropriate CD25-FITC and Foxp3-PE antibodies with the mouse regulatory T-cell staining kit (eBiosciences) according to the manufacturer’s instructions. The frequency of CD4^+^CD25^+^Foxp3^+^ Treg cells was tested on FACs. Data analysis was carried out in the FlowJo.

### Statistical analysis

One-way analysis of variance (ANOVA) tests (Tukey’s Multiple-Comparison Test) were performed to analyze the differences among multiple experimental groups. All values are expressed as mean ±SD. *P*< 0.05 is believed to be statistically significant. The statistical analyses were performed using Prism 5.0 software.

## Results

### WPCD promoted the maturation and function of BM-DCs *in vitro*

**Fig 1:**
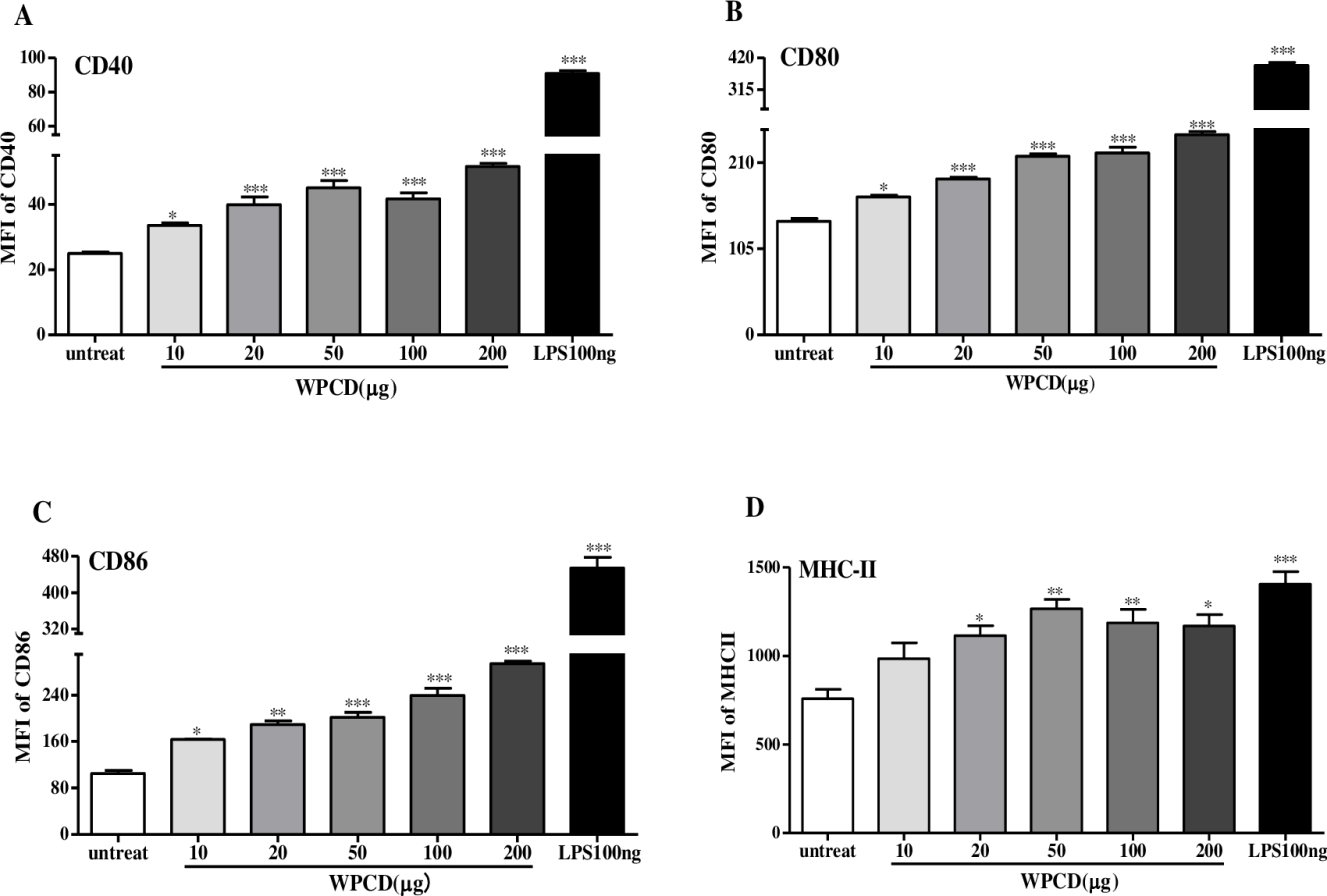
Effects of WPCD upon DCs phenotypic maturation *in vitro*. BM-DCs from C57BL/6 mice were treated with different concentrations of WPCD for 12 h. LPS was used as the positive control. Then, the expression levels of CD11c, CD40, CD80, CD86, and MHC-II in cells were analyzed. Phenotypic maturation of BM-DCs was induced by PBS, LPS and WPCD. The mean fluorescence intensity values (MFI) of CD40 **(A)**, CD80 **(B)**, CD86 **(C)**, and MHC-II **(D)** were shown. Data represent mean ± SD (n = 3) from 3 independent experiments. *P < 0.05, ** *P* < 0.01, *** *P* < 0.001 compared with the untreated DCs group.

Adaptive immunity is determined by the activation of DCs, including the types of co-stimulatory molecules and cytokines [29, 30]. Initially, we investigated whether WPCD could promote the expression of co-stimulatory molecules. According to different doses of WPCD, BM-DCs from C57BL/6 were administered. The expression levels of CD11c, CD86, CD80, CD40, and MHC-II in cells were analyzed (Fig 1). The cells gated with SSC and FSC showed that the treatment with different doses of WPCD did not change the morphology of BM-DCs (data not shown). The expression levels of CD86, CD80, and CD40 were significantly up-regulated in a dose-dependent manner compared to the untreated group and reached the plateau under the dose of 20 μg/mL of WPCD, but the difference was not significant compared with LPS group (Fig 1A–1C). The expression level of MHC-II was significantly increased to its maximum value under the dose of 50 μg/mL (Fig 1D). The analysis results of cellular surface markers demonstrated that DCs treated with LPS or WPCD showed the significantly increased expression levels of CD86, CD80, CD40, and MHC-II and the promoted phenotypic maturation.

**Fig 2:**
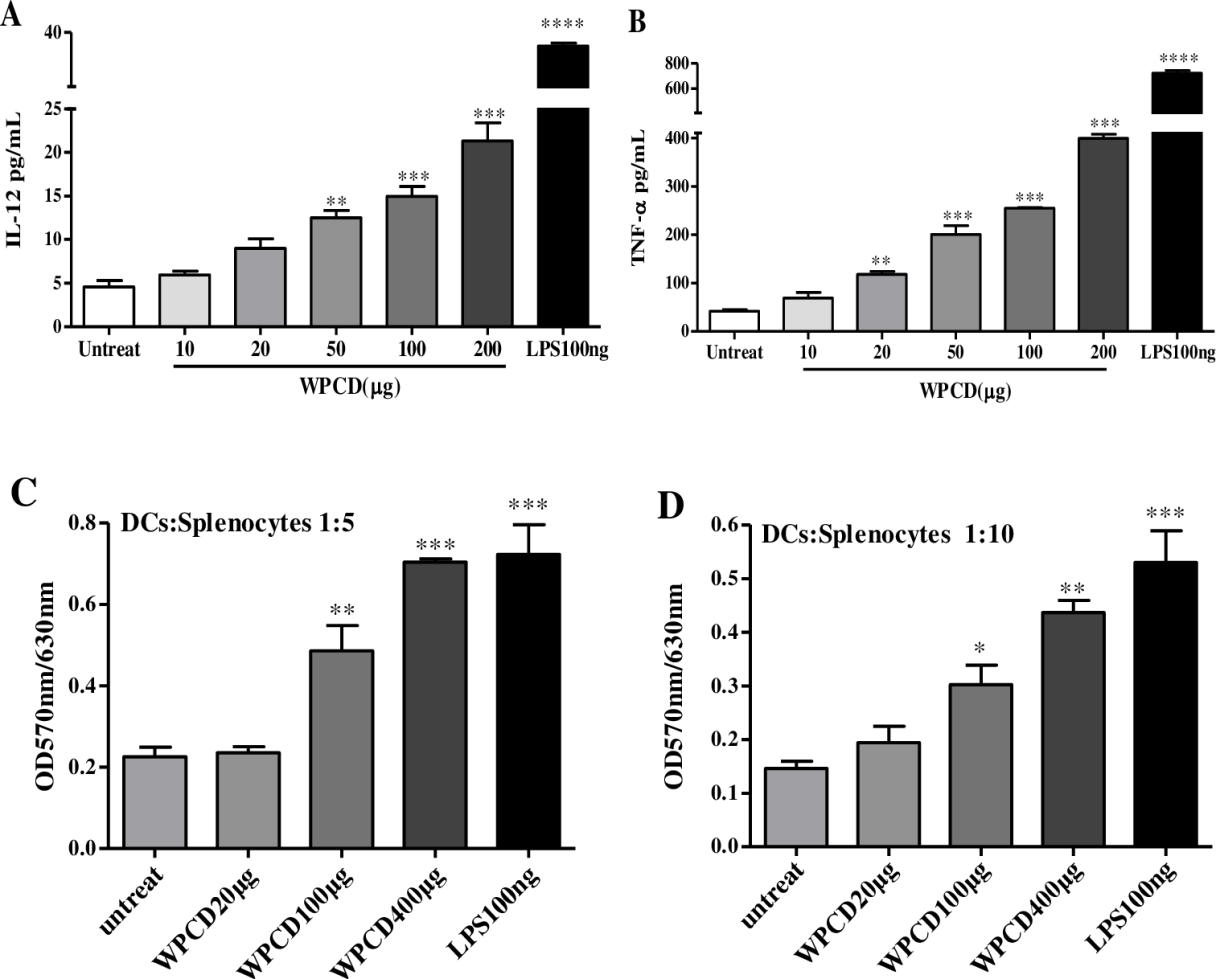
Effects of WPCD on cytokine production and functional activation of DCs *in vitro*. After DCs were treated with different concentrations of WPCD for 12 h, the supernatant was collected and the productions of IL-12 and TNF-α were detected by ELISA. The concentrations of IL-12 **(A)** and TNF-α **(B)** were shown. An allogenic T cell proliferation of WPCD-treated DCs was analyzed by MLR. Allogenic T cells were co-cultured for 3 days at the indicated ratio with DCs which had been cultured for 12 h in triplicate **(C and D)**. MTT was added into the cultures for final 4-h treatment and the T cell proliferation was then determined by ELISA reader. Data represent mean ±SD (n = 3) from 3 independent experiments.*P < 0.05, ** *P* < 0.01, *** *P* < 0.001 compared with the untreated DCs group.

Some adjuvants could increase co-stimulatory molecules on DCs or directly induce the secretion of cytokines. IL-12 and TNF-α is the major cytokines for activating Th1 immune response. We analyzed the yields of cytokine in BM–DCs upon WPCD treatment. After BM–DCs were treated with different contents of WPCD for 12 h, the supernatant was collected to detect the contents of IL-12 and TNF-α with ELISA kit. WPCD could dose-dependently increase the productions of IL-12 (Fig 2A) and TNF-α (Fig 2B). The results demonstrated that WPCD could induce the functional maturation of DCs.

In an immune response, it is necessary to functionally activate DCs. Then, the higher level of allogenic T cell proliferation was induced by fully mature DCs. Therefore, the functional response of DCs to WPCD was investigated by MLR. The allostimulatory capability of WPCD-induced DCs, similar to LPS, was enhanced dramatically in a dose-dependent manner at the indicated ratio (Fig 2C and 2D). The results indicated that WPCD improved antigen presentation and optimized T-cell response by MLR *in vitro*.

### WPCD promoted maturation of DCs via TLR4 pathway

**Fig 3:**
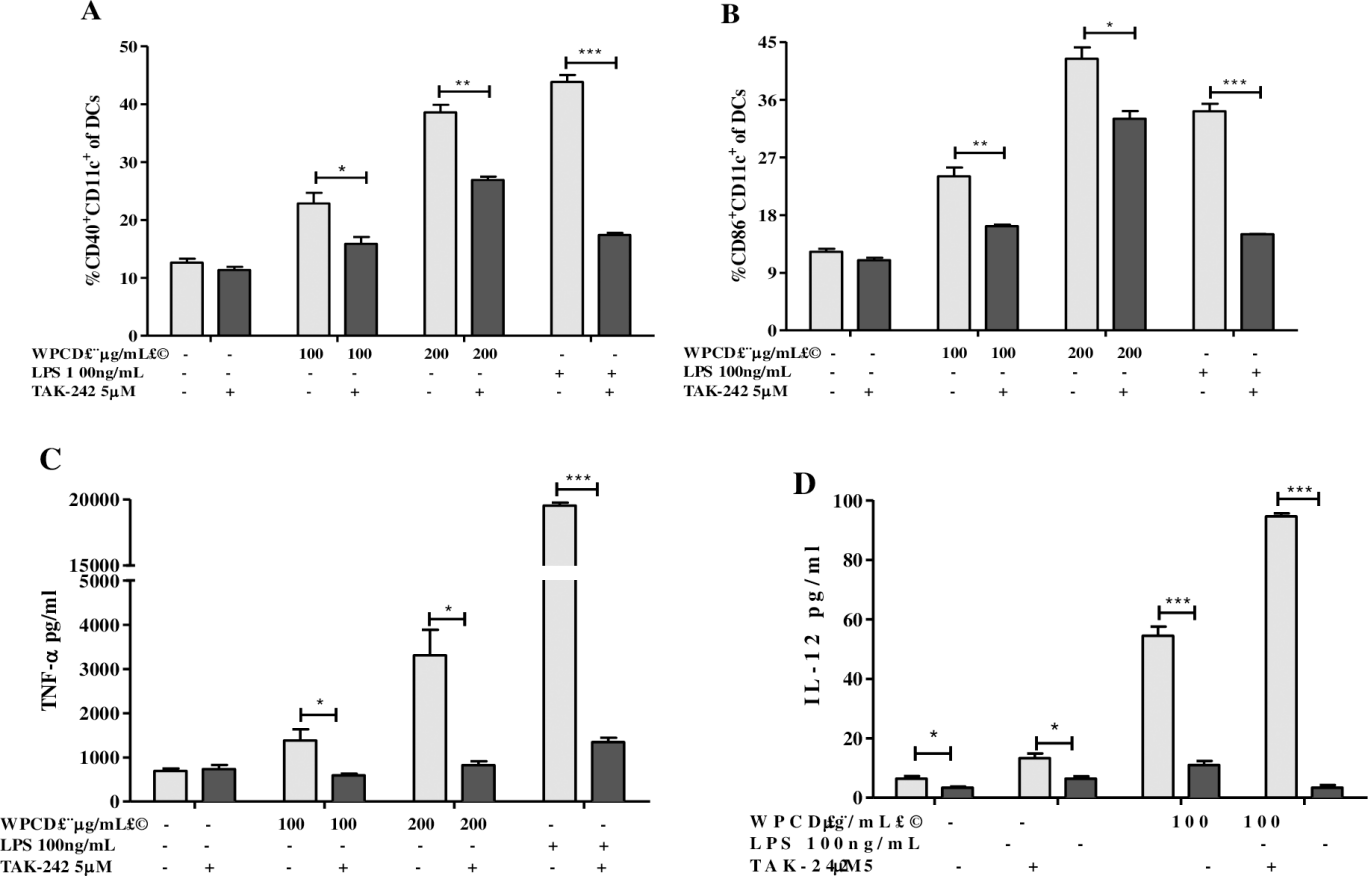
Effects of WPCD on BM-DCs function through TLR4-mediated pathway. After pretreatment with 5 μM of TAK-242 for 1 h, BM-DCs were treated with 100 ng/mL LPS or 100 and 200 μg/mL WPCD for 12 h in the presence or absence of Golgi Stop. Then CD40, CD86, TNF-a and IL-12 expressions were detected by FACs or ELISA. The MFI values of CD40 **(A)** and CD86 **(B)**, the yields of TNF-a **(C)**, and IL-12 **(D)** were shown. Data represent mean ± SD (n = 3) from 3 independent experiments.*P < 0.05, **P < 0.01, ***P < 0.001 compared with the untreated DCs group.

It could be inferred from the above results that BM-DCs activated by WPCD indicated similar DCs surface molecule expressions to LPS (a TLR4 ligand). We thus hypothesized that BM-DCs are activated by WPCD via TLR4 pathway. After BM-DCs were pretreated with TAK-242 (TLR4 inhibitor) and co-cultured with WPCD and LPS for 12 h, the expression of CD40, CD86, TNF-a or IL-12 were detected by FACs or ELISA. As shown in Fig 3A and 3B, the TAK-242 treatment resulted in the significantly down-regulated expressions of CD40 and CD86 induced by LPS or WPCD, and cytokine production of IL-12 or TNF-a were also significantly decreased (Fig 3C and 3D). The results indicated that WPCD could activate DC maturation through the TLR4 pathway.

### WPCD enhanced humoral and cellular immunity

**Fig 4:**
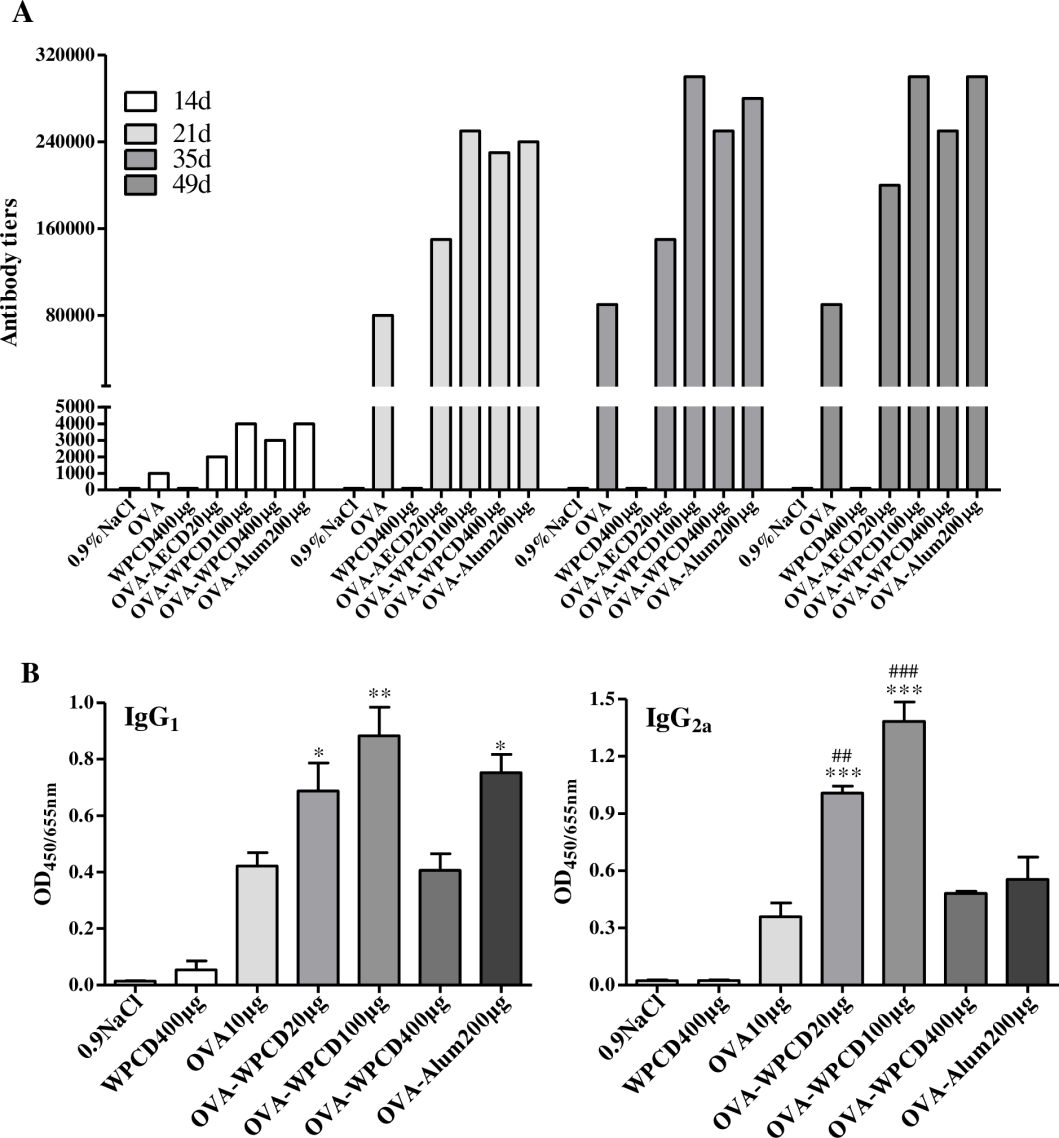
Effects of WPCD on OVA-specific IgG and IgG subclass. Effects of WPCD on OVA-specific IgG, IgG_1_ and IgG_2a_ antibody in serum were assessed by ELISA. IgG titer on Days 14, 21, 35, and 49 after the first vaccination **(A)**, the antibody levels of IgG_1_ and IgG_2a_ on Day 35 after the first vaccination **(B).** Data represent mean ± SD (n = 5) from 3 independent experiments. *P< 0.05, **P< 0.01, ***P< 0.001 compared with the OVA group; ^##^*P*< 0.01, ^###^*P* < 0.001 compared with the OVA/Alum group.

For the purpose of comparison, we evaluated the effects of WPCD on the elicitation of humoral immune response in OVA-immunized mice. Before vaccination and on Days 14, 21, 35, and 49 after the first vaccination, blood was collected to detect IgG titer and IgG subclasses serum by ELISA. The IgG antibody response to OVA increased with the increase in the dose of WPCD administration (100 and 400 μg) in a dose-dependent manner (Fig 4). The optimal concentration was 100 μg/mL of WPCD and increased IgG responses by two times compared to the OVA alone on Days 21, 35 and 49 (Fig 4A). Considerable increases in OVA-specific IgG_1_ and IgG_2a_ antibody levels under 20 and 100 μg of WPCD administration were obtained compared with OVA group in serum on Day 35 (Fig 4B). Meanwhile, Alum promoted only the expression levels of OVA-specific IgG and IgG_1_ antibodies in the immunized mice. WPCD alone did not trigger OVA-specific antibody. The above results obviously indicated that WPCD generated the higher antibody titers and the more balanced Th1/Th2 responses than Alum.

**Fig 5:**
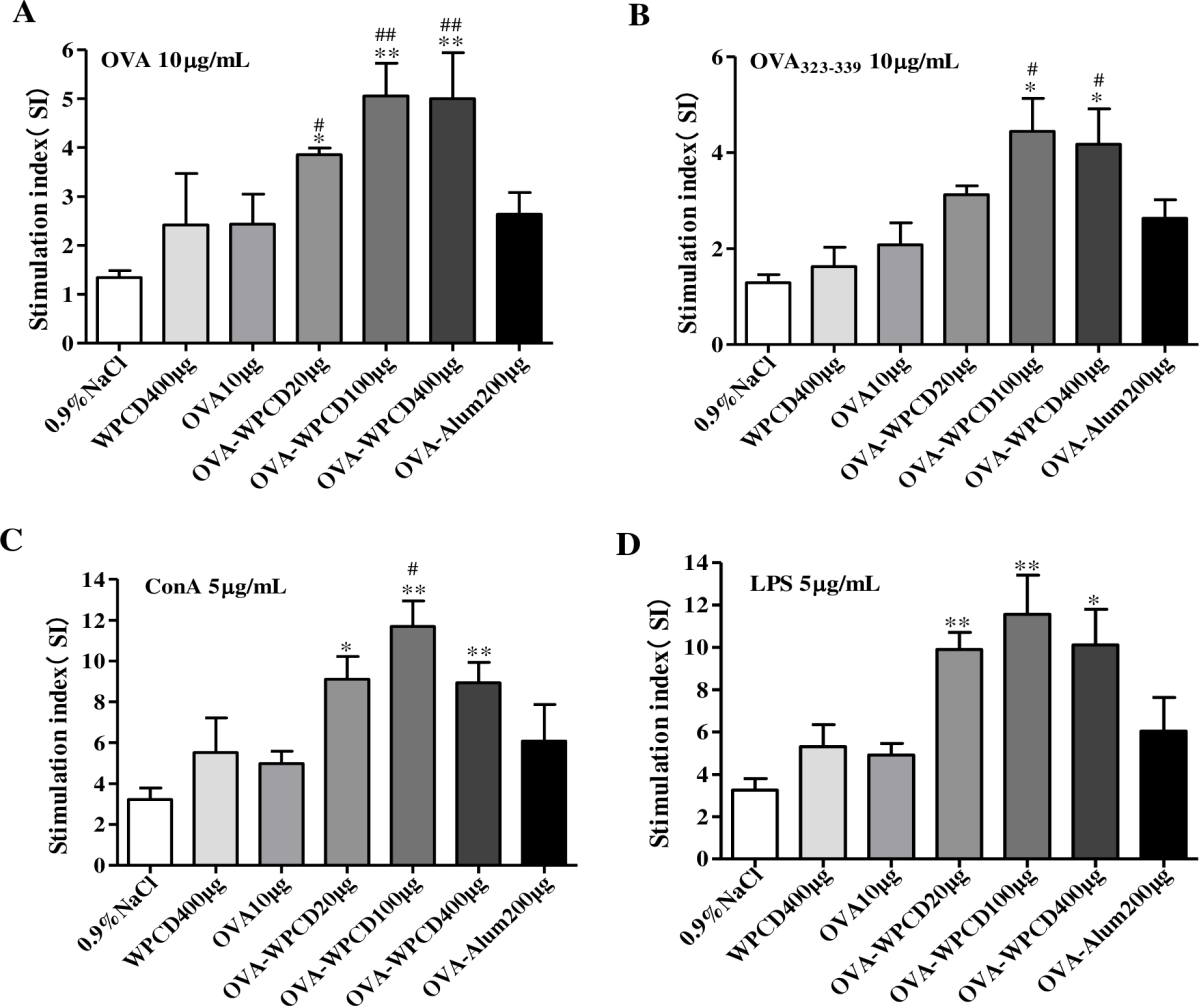
Effects of WPCD on the splenocyte proliferation. Single splenocyte was isolated from mice immunized on day 21 after the first vaccination and splenocyte proliferation was determined by the MTT assay and expressed as SI. OVA-antigen stimulated splenocyte proliferation **(A)**. OVA^323-339^-antigen stimulated splenocyte proliferation **(B).** ConA-mitogen stimulated splenocyte proliferation **(C)**. LPS-mitogen splenocyte proliferation **(D)**. Data are expressed as mean ± SD (n = 5) from 3 independent experiments. *P < 0.05, ** *P* < 0.01 compared with the OVA group; ^#^*P* < 0.05, ^##^*P* < 0.01 compared with the OVA/Alum group.

Splenocyte proliferation is another indicator of cellular immunity. ConA stimulates T-cells and LPS stimulates B-cell proliferation. On Day 21 after the first vaccination, single splenocyte suspension was obtained and respectively stimulated with OVA (10 μg/mL), OVA^323-339^ (10 μg/mL) peptide, ConA (5 μg/mL) and LPS (5 μg/mL) for 48 h. Then the proliferation of T cell was measured by MTT method. WPCD could significantly promote OVA-antigen, Con A-mitogen and LPS mitogen-stimulated splenocyte proliferation in the mice immunized with OVA and WPCD (Fig 5) and the optimal concentration of WPCD was 100 μg/mL. These data indicated that WPCD as a vaccine adjuvant in OVA-immunized mice could more effectively induce the activation of T-cells and B cells than Alum.

**Fig 6:**
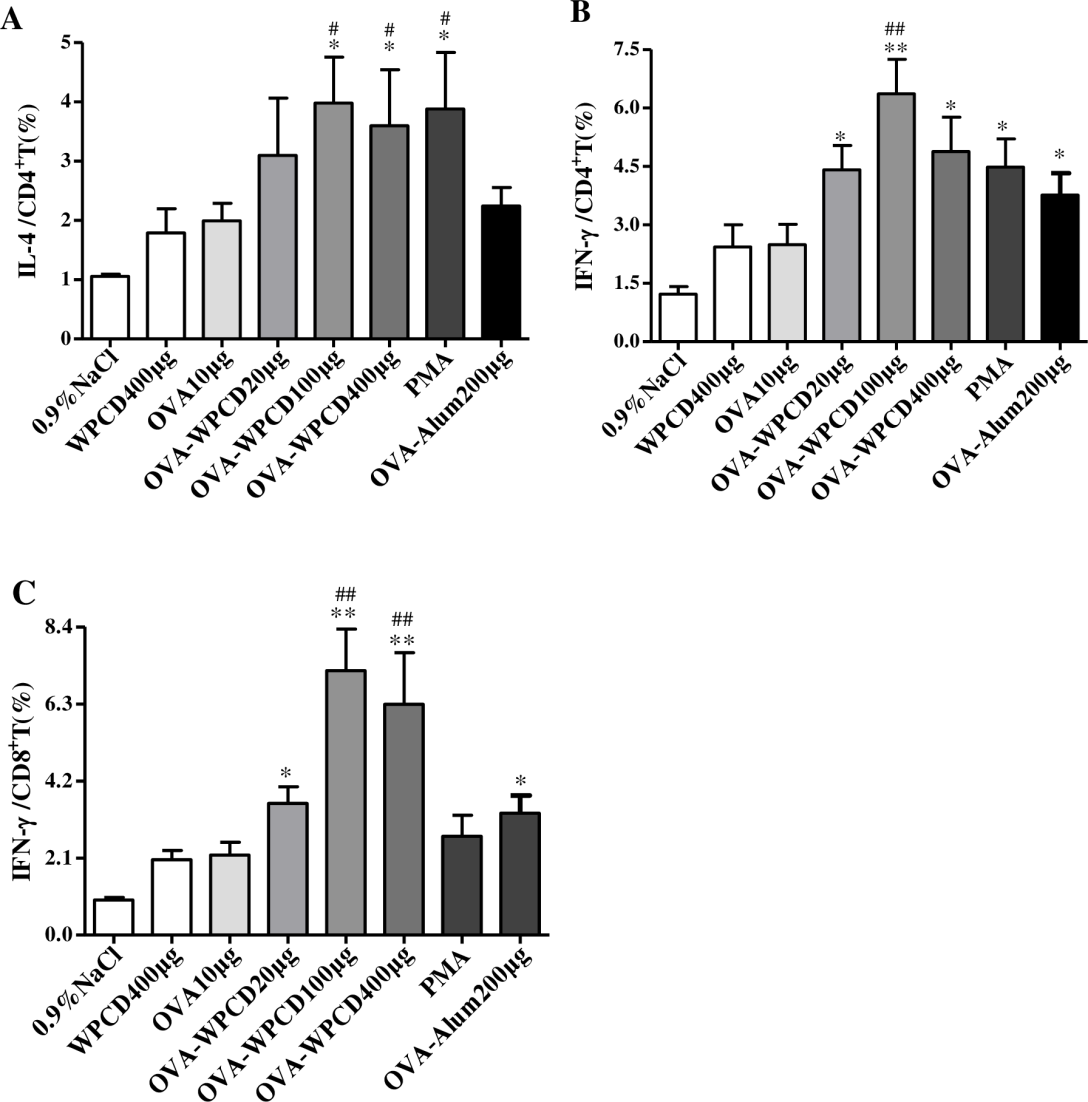
Effects of WPCD on IL-4 and IFN-γ production from splenocytes. On Day 21 after the first vaccination, single splenocyte was isolated from mice immunized and co-cultured with OVA (10 μg/mL) for 12 h. Productions of IFN-γ and IL-4 were determined by FCA_S_. IL-4 production in CD4^+^ T cells **(A)**, IFN-γ production in CD8^+^ T cells **(B)**, and INF-γ production in CD8^+^ T cells **(C)**. Data represent mean ±SD (n = 5) from 3 independent experiments. * *P* < 0.05, ** *P* < 0.01 compared with the OVA group; #P < 0.05, ##P < 0.01 compared with the OVA/Alum group.

All tested WCPD adjuvants formulated with OVA vaccines generated equally strong humoral and cellular immunity (Figs 4 and 5). On the basis of these data, to further evaluate WPCD influences on OVA-specific T helper (Th) cell response, we further analyzed cytokine expressions in CD8^+^ and CD4^+^ T cells by Flow cytometry (Fig 6). On Day 21 after the first vaccination, single splenocyte of mice was isolated and then co-cultured with the OVA (10 μg/mL). IL-4 yield in CD4^+^ T cells in the group administered with 100 μg OVA/WPCD was significantly higher than that in OVA/Alum group and OVA group and similar to that in PMA (Fig 6A). Moreover, IFN-γ yield in CD8^+^ and CD4^+^ T cells was also significantly enhanced in the mice administered with OVA/WPCD (20, 100, and 400 μg) (Fig 6B and 6C). Compared with Alum adjuvant, WPCD showed the better capability of inducing IL-4 and IFN-γ secretions from T cells.

### WPCD stimulated maturation of DCs and decreases Treg frequency *in vivo*

**Fig 7:**
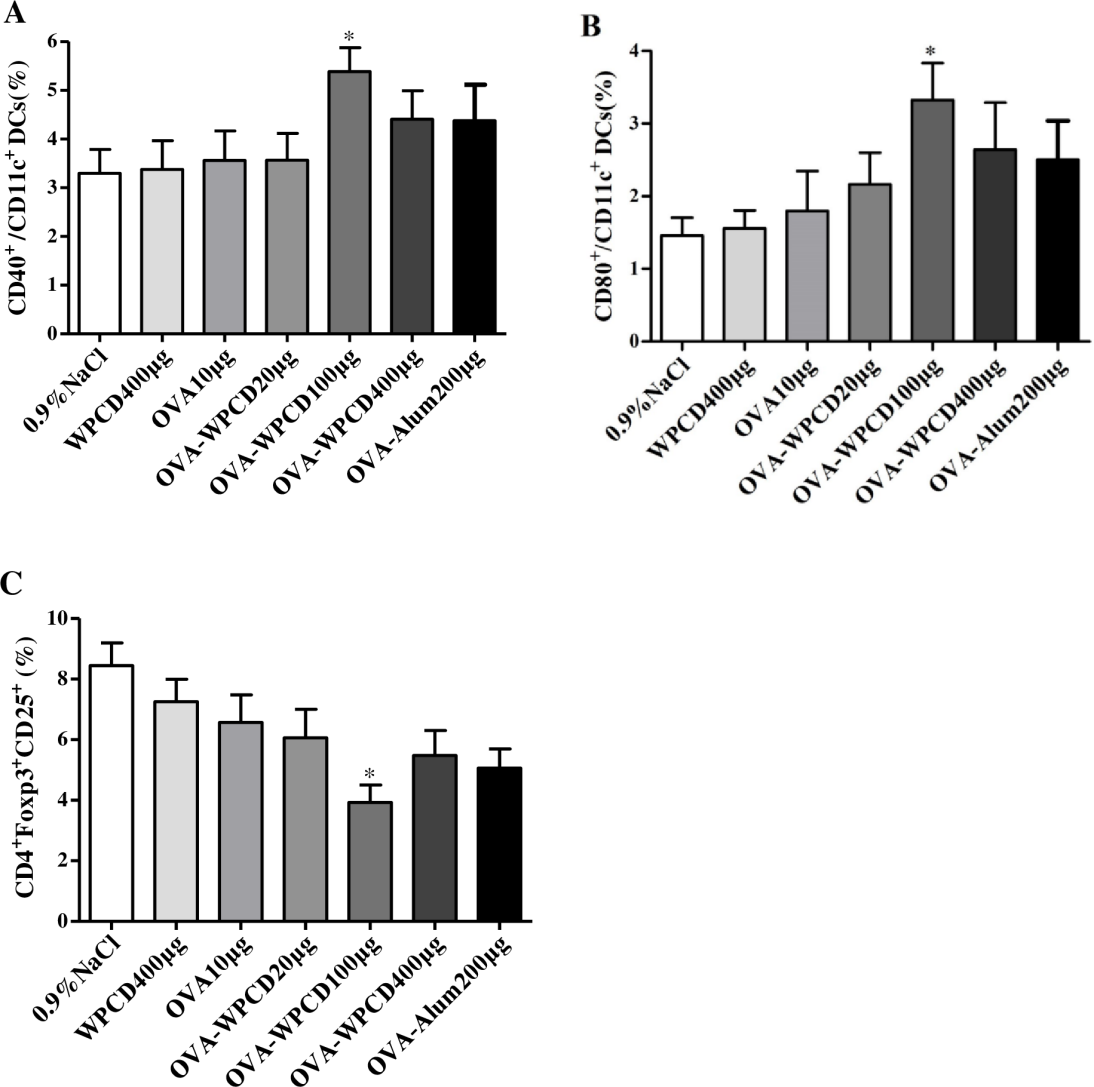
Effects of WPCD on DCs maturation and Treg frequency. On Day 3 or 21 after the first vaccination, cells were separated from the spleen of each group mice and the expression levels of CD11C^+^CD40^+^ and CD11C^+^CD80^+^ on DCs as well as the frequency of CD4^+^CD25^+^Foxp3^+^ was measured by FCA_S_. The expressions of CD11C^+^CD40^+^ **(A)** and CD11C^+^CD80^+^ **(B)** on DCs in the spleen on Day 3 after the first vaccination. The frequency of CD4^+^CD25^+^Foxp3^+^ Treg in the total CD4^+^ T cells in the spleen on Day 7 after the second vaccination **(C)**. Data represent mean ± SD (n = 5) from 3 independent experiments. *P < 0.05 compared with the OVA group.

DCs are recognized as the most potent APCs involved in initiating primary immune responses. Thus, on Day 3 after the first vaccination, we also investigated the effects of WPCD on CD40 and CD80 on DCs in the spleen in mice (Fig 7A and 7B). The mice in 100-μg OVA/WPCD group produced the significantly higher levels of CD40 and CD80 on DCs than those in the OVA/Alum group. These data showed that WPCD could activate DCs and induce DCs maturation in mice.

Treg cells can balance the tolerance and immune responses. To further investigate how WPCD modulated the immune response, Treg cells in the spleen in mice were stained with mouse regulatory T cell staining kit. On Day 21 after the first vaccination, a decreased frequency of CD25^+^Foxp3^+^ Treg cells was observed in the total CD4^+^ T cells (Fig 7C). Compared with the OVA and OVA/Alum groups, WPCD group showed the significantly reduced Treg frequency.

### Safety evaluation of WPCD in mice

In order to estimate the oral acute toxicity, we carried out the acute toxicity test. The mice orally administered with 5000 mg/kg body weight showed no abnormal behavior or side effects and no mortality was found in the toxicity evaluation test. No significant difference in body weight gain, Thymus index and Spleen index was recorded among various groups of the mice administered with different doses of WPCD and had no significant difference (Table 1). No mortality was observed after 14 days. Therefore, the LD_50_ value of WPCD was more than 5000 mg per kg body weight.

**Table 1:** Evaluation of the oral acute toxicity of WPCD.

Group(mg/kg)	Day 1	Day 7	Day 14	Thymus index	Spleen index
0.9%NaCl	18.5±1.80	23.3±1.89	24.8±1.75	3.98±1.44	3.69±1.49
WPCD50	19.2±1.75	23.1±1.77	24.3±1.75	3.43±0.65	2.78±0.84
WPCD 500	18.1±1.37	21.9±1.83	23.5±1.52	2.91±0.59	2.71±0.78
WPCD 5000	18.8±1.55	23.2±1.61	24.1±1.25	3.39±0.54	2.72±1.24
Alum 250	19.6±1.74	24.6±2.27	25.4±1.94	3.46±1.63	2.99±1.33

Values are means ± SD (n = 8). No significant difference in body weight gain, Thymus index or Spleen index (*P*>0.05).

To test whether WPCD had negative effects on the growth of the mice, before and after subcutaneous vaccination, the body weight was respectively determined for each mouse (Table 2). In the subsequent observation of the mice, side effects or abnormal behaviors were not observed. Besides, the body weight of the mice administered with WPCD and the mice administered with saline solution or OVA/Alum showed no significant difference. These observation results demonstrated that the administration of WPCD was safe.

**Table 2:** Effects of subcutaneous administration of WPCD on the mean body weight.

Groups	Day 0	Day 14	Day 28	Day 42
0.9% NaCl	16.74±0.55	24.28±0.98	28.30±1.43	30.36±0.99
WPCD μg	16.02±0.46	26.82±0.65	30.31±0.59	31.16±2.84
OVA	18.04±0.54	25.87±1.19	28.55±1.02	30.08±0.81
OVA/WPCD 20 μg	18.36±0.15	25.80±0.91	28.34±0.71	29.18±0.96
OVA/WPCD 100 μg	15.20±0.89	26.22±1.73	28.44±1.61	30.94±1.47
OVA/WPCD 400 μg	15.56±1.05	25.68±0.87	29.44±0.81	30.66±0.65
OVA-Alum200 μg	16.24±0.70	25.42±1.79	28.77±1.35	29.66±0.65

Before and after subcutaneous vaccination, the body weight was respectively determined for each mouse. The body weight of the mice administered with WPCD and the mice administered with saline solution or OVA/Alum showed no significant difference (*P*>0.05).

## Discussion

Adjuvants are key components in vaccines [31]. Due to the advances in genomics and proteomics, more and more recombinants and synthetic vaccine molecules are identified. Therefore, more adjuvants and formulations are required. Some potent adjuvants are generally related to the increased toxicity, for example FCA. Therefore, it is necessary to find a safe formulation containing different synergistic components eventually driving the desired immune response. Polysaccharides from Chinese herbs are nontoxic and show no significant side effect [32,28]. A safe adjuvant is required for a certain vaccine.

*Cistanche deserticola* is an important tonic herb and widely exists in arid lands and warm deserts in the northwest of China. *Cistanche deserticola* is widely concerned due to its bioactivities including immunomodulatory, antioxidative, antibacterial and antitumor effects [20,21]. Some progresses have been made in structural characterization and immunological activity of *Cistanche deserticola*, which is a good candidate for developing adjuvants. We experimentally evaluated the adjuvant effects and mechanism of WPCD *in vitro* and *in vivo*. Subcutaneous administration of WPCD significantly promoted humoral and cellular immune responses by increasing serum antibodies and lymphocyte proliferation, enhancing the expression of cytokines, up-regulating the DC maturation, and down-regulating the frequency of CD4^+^CD25^+^ Foxp3^+^ Treg cells.

DCs are key APCs for priming naive T cells. The activation of DCs is important for adjuvants. DCs, especially murine marrow-derived DCs, are often used to evaluate adjuvants and vaccines. Flow cytometry can distinguish the cells which have been activated after capturing an antigen from surrounding cells. In FCM analysis, the degree of aggregation of stimulated cells is an indirect indicator of evaluating safety of immunomodulators [3]. Thus, WPCD adjuvant activity data in DCs *in vitro* may provide valuable information for animal models. Based on BM-DC model, the expression levels of MHC-II, CD86, CD80, and CD40, cytokine production, and allogenic T cell proliferation were detected in the optimum WPCD concentration range. The expression levels of MHC-II, CD86, CD80, and CD40 were up-regulated in BM-DCs and the yields of TNF-a and IL-12 were increased in a dose-dependent manner. The allogenic T cell proliferation was observed. The morphology of BM-DCs was not changed. By inducing BM-DCs activation and the secretion of inflammatory cytokines *in vitro*, WPCD adjuvant might significantly increase the amount of antigen and the amount of APCs and stimulate T cells to secrete IFN-γ, which contributes to the enhancement of immunomodulatory activity.

Various Chinese herbaceous polysaccharides are capable of activating the immune system and possess excellent adjuvant abilities through stimulating DCs maturation by TLR4 pathway [33,28]. Thus, we assume that TLR4 participates in the signaling pathway of WPCD-induced DCs maturation. As expected, TLR4 inhibitor treatments resulted in a significant decrease of TNF-a and IL-12. Furthermore, the inhibition of TLR4 pathway also hindered the expression of CD 40 and CD80 on DCs, indicating that the maturation of DCs was dependent on TLR4. Therefore, it is obvious that TLR4 participates in the WPCD-induced DCs maturation.

The optimum dose of adjuvants and vaccine formulation is empirically determined in many experiments. In order to explore the dose-response relationship of WPCD adjuvant and OVA antigens in mice, we selected different WPCD doses based on previously reported data from BM-DCs *in vitro* to subcutaneously administer ICR mice twice. We found that WPCD significantly increased the yield of OVA-specific antibody and induced a balanced Th1/Th2 immune response with an enhancement of IgG_1_ and IgG_2a_ levels. Especially, the IgG_2a_ level was higher than that treated with Alum in the optimum dose. Therefore, WPCD resulted in the higher levels of specific antibody and higher efficacy with the lower injections.

New vaccines require the induction of strong cellular responses, which involve antibodies, T helper (Th) cells, and cytotoxic T lymphocytes (CTLs). Therapeutic vaccine aims to induce stronger T cell responses. The ideal adjuvant enhances the potency of the vaccine and promotes cell-mediated immunity without causing toxic effects [34]. Among the observations in immunized mice models, WPCD led to an increase in OVA-specific and non-specific splenocyte proliferation compared with Alum. Some adjuvants up-regulate cytokines and the immune system. For example, saponins may stimulate cell-mediated immune responses to an antigen which normally induces only antibodies. We selected IL-4 and IFN-γ as the indicators to indirectly evaluate the levels of immunities in mice. WPCD could induce more IL-4 and IFN-γ secretions than Alum. Thus, strong helper T-cell responses and cytokine secretions were observed as a result of WPCD vaccination, suggesting that WPCD could more efficiently stimulate lymphocytes to secret the Th1-type cytokine and the Th2-type cytokine than Alum.

Adjuvants influencing antigen presentation can affect complex immune processes. Treg cells can modulate Th1 and Th2 responses [35]. An appropriate dose of WPCD may promote the maturation of DCs in mice via increasing the expression levels of CD80 and CD40 and amplify the capability of OVA antigen presentation in the early immune response. The experimental results of the DCs activation and Treg frequency demonstrated that WPCD induced maturation of DCs and decreased Treg frequency in the spleen in mice. These results proved that WPCD enhanced the efficacy of OVA vaccines by increasing targeting antigen-presenting cells and promoting T-cell specific activation.

In the development of adjuvants, it is difficult to selectively induce the appropriate immune response against corresponding infection. Suitable adjuvant should have low side effects and toxicity to human beings or animals. Thus, WPCD safety should be considered, including immediate and long-term side effects. In this study, we explored acute toxicity of WPCD and the negative effect of WPCD on the growth performance of mice for 110 days. The results of acute toxicity of WPCD showed that body weight, thymus index, or Spleen index had no significant difference. The LD50 value of WPCD was more than 5000 mg per kg body weight. Throughout the experimental subsequent observation of the mice, no side effect or abnormal behavior in mice was found. These results indicated that WPCD was safe.

In conclusion, in this study, WPCD, a crude polysaccharide extracted from the *C*. *deserticola* from Xinjiang, exhibited some properties of adjuvants. For example, WPCD enhanced both Th1 and Th2 responses by activating DCs, increasing antibody responses, improving cytokine production. Moreover, we explored the mechanism of WPCD efficacy by analyzing DCs maturation and function via TLR4 pathway *in vitro*. In the mice only treated with WPCD, no obvious side effect was observed. Hence, WPCD possessed the higher immunostimulatory activity in cellular and humoral immune responses. Adjuvants are a mixture of several compounds. A mixture of several crude extracts may have greater beneficial effects than a single plant extract. It is necessary to systematically explore WPCD extracts, test their efficacy and safety, and elucidate the mechanisms of their effects.

## Supporting information

S1 File ARRIVE Checklist. (DOCX)
