# Supplementary material for: Immunostimulatory activity of water-extractable polysaccharides from Cistanche deserticola as a plant adjuvant in vitro and in vivo
Source: PLoS One. 2018 Jan 23;13(1):e0191356. doi: 10.1371/journal.pone.0191356 (PMC5779666; doi:10.1371/journal.pone.0191356)

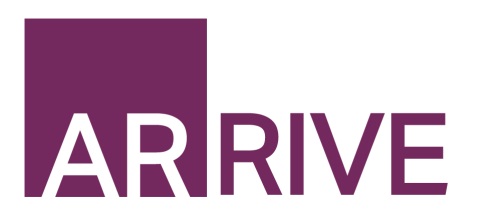


The ARRIVE Guidelines Checklist

Animal Research: Reporting In Vivo Experiments

Carol Kilkenny^1^, William J Browne^2^, Innes C Cuthill^3^, Michael Emerson^4^ and Douglas G Altman^5^

*^1^The National Centre for the Replacement, Refinement and Reduction of Animals in Research, London, UK, ^2^School of Veterinary Science, University of Bristol, Bristol, UK, ^3^School of Biological Sciences, University of Bristol, Bristol, UK, ^4^National Heart and Lung Institute, Imperial College London, UK, ^5^Centre for Statistics in Medicine, University of Oxford, Oxford, UK.*

|  | | ITEM | RECOMMENDATION | Section/ Paragraph |
| --- | --- | --- | --- | --- |
| 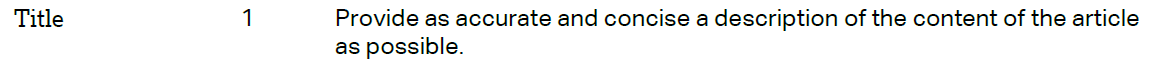 | | | Title |  |
| 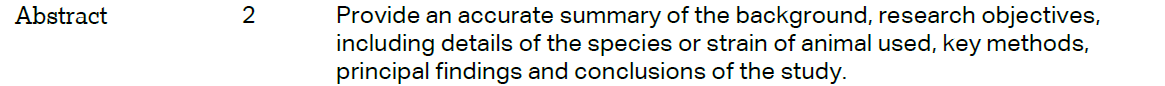 | | | Abstract |  |
| INTRODUCTION | | |  |  |
| 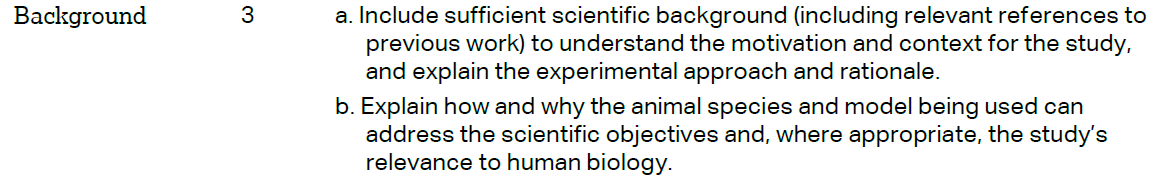 | | | Paragraph 1-5  Paragraph 1-2 |  |
| 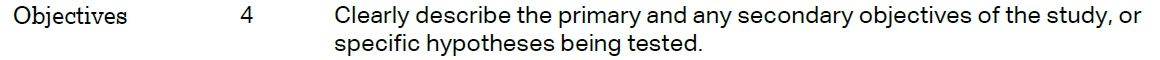 | | | Paragraph 5 |  |
| METHODS | | |  |  |
| 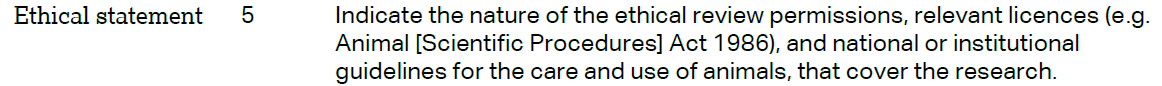 | | | Paragraph 1 |  |
| 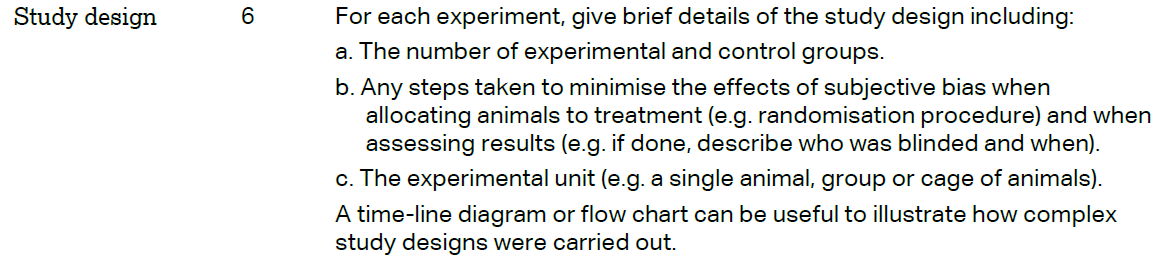 | | | Paragraph 3-5, 8-9  Paragrap,3-5,, 8-9  Paragraph 8-9 |  |
| 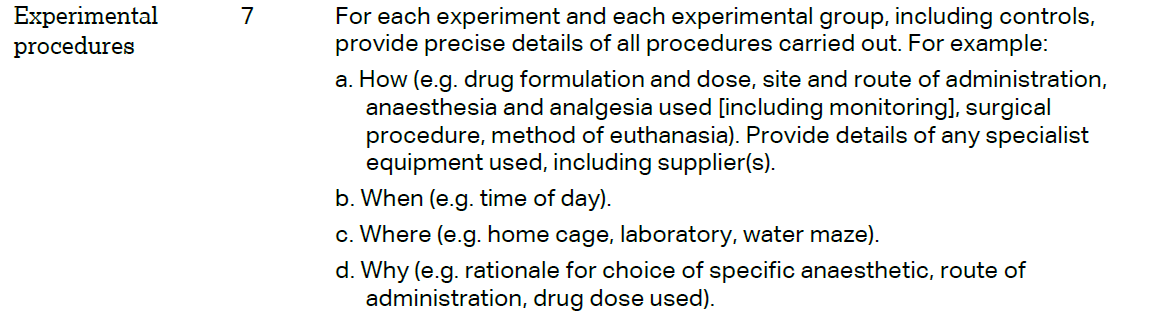 | | | Paragraph 2-7,10-14 |  |
| 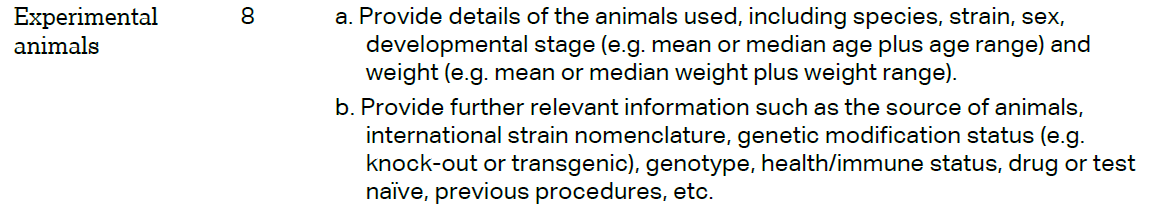 | | | Paragraph 1 |  |

The ARRIVE guidelines. Originally published in *PLoS Biology*, June 2010^1^

| 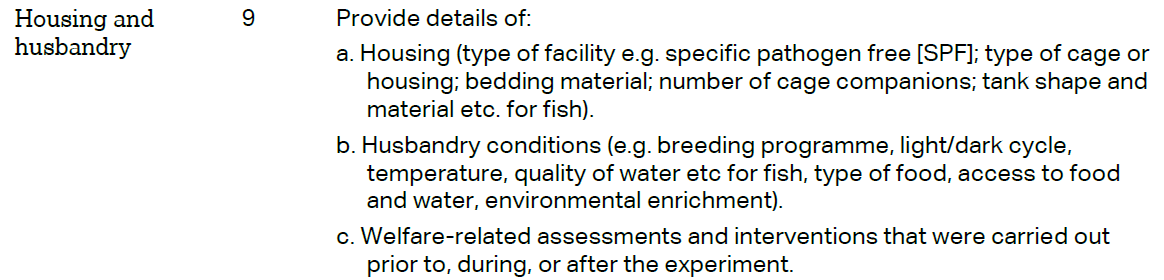 | Paragraph 1 | |
| --- | --- | --- |
| 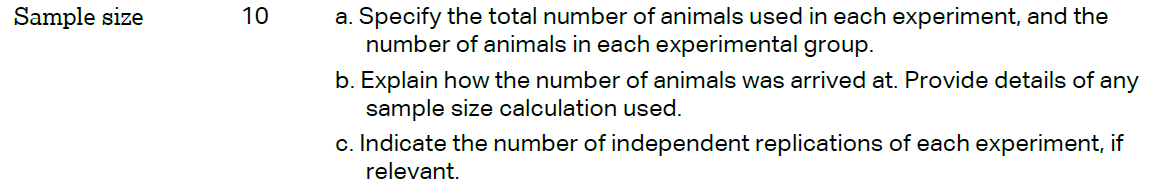 | Paragraph 8-9 | |
| 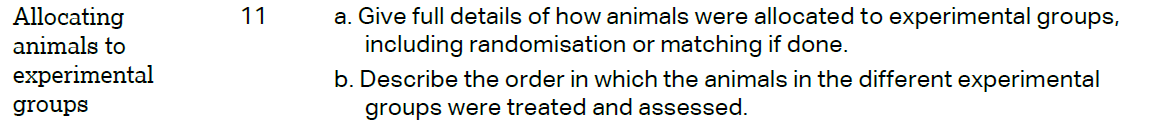 | Paragraph 8-9 | |
| 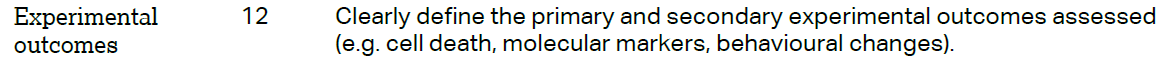 | Paragraph 15 | |
| 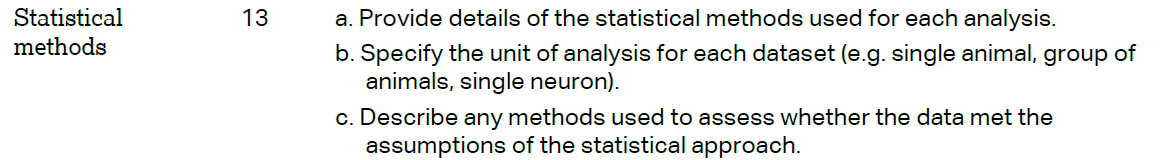 | Paragraph 15 | |
| RESULTS |  | |
| 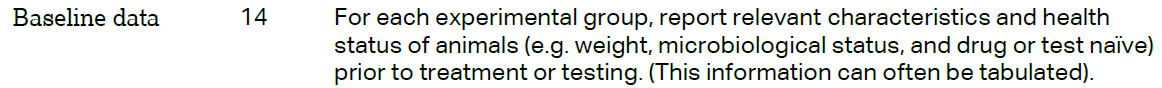 | Methods Paragraph 1 | |
| 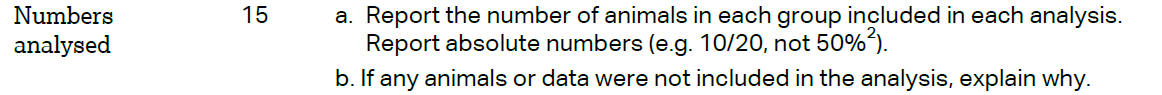 | Methods Paragraph 8-9 | |
| 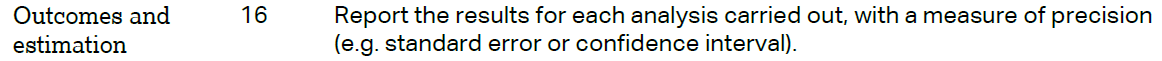 | Paragraph 1-9  Fig 1-7 | |
| 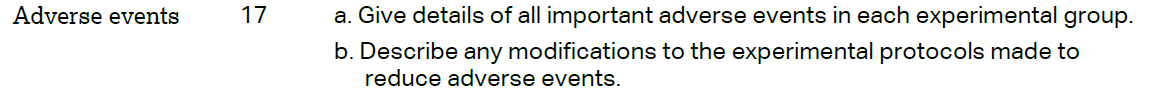 | Paragraph 10-11 | |
| DISCUSSION |  | |
| 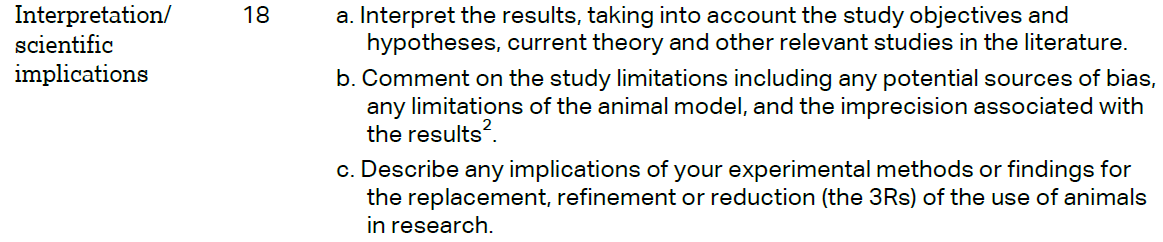 | Throughout  Paragraph1,8 | |
| 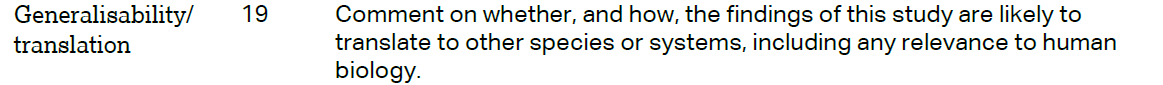 | Paragraph1,9 | |
| 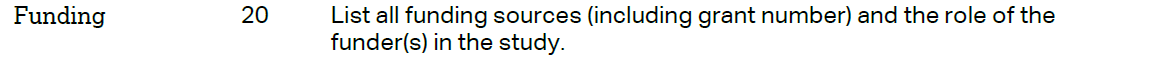 | |  |


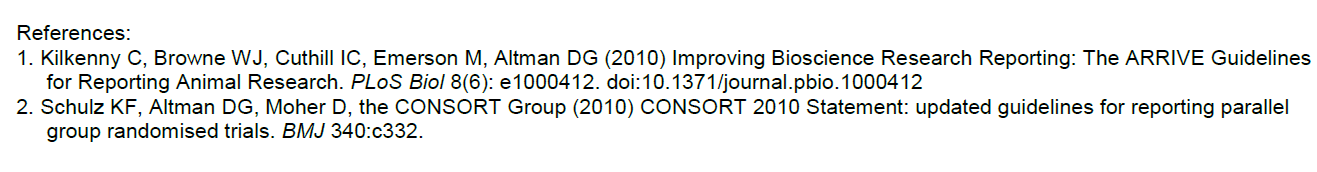

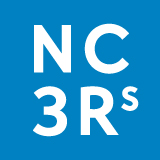

Supplement: S1 File — (DOCX) [file pone.0191356.s001.docx]
